# Clinical Applications of Hyperpolarized Magnetic Resonance Imaging in Brain Tumors: Current Evidence and Future Opportunities

**DOI:** 10.3390/cancers18152462

**Published:** 2026-07-31

**Authors:** Riccardo Serra, Siddharth R. Shah, Adarsha P. Malla, Tina Wang, Alexander Ksendzovsky, Dirk Mayer, Eli E. Bar, Graeme F. Woodworth

**Affiliations:** 1 Department of Neurosurgery, University of Maryland School of Medicine, Baltimore, MD 21201, USA; rserra@som.umaryland.edu (R.S.); siddharth.shah@som.umaryland.edu (S.R.S.); apmalla@som.umaryland.edu (A.P.M.);; 2 Brain Tumor Program, Greenebaum Comprehensive Cancer Center, University of Maryland, Baltimore, MD 21201, USA; 3 Department of Diagnostic Radiology and Nuclear Medicine, University of Maryland School of Medicine, Baltimore, MD 21201, USA

**Keywords:** hyperpolarized MRI, brain tumors, glioma, glioblastoma, metabolic imaging, carbon-13 MRI, tumor metabolism, precision neuro-oncology

## Abstract

Brain tumors exhibit extensive metabolic reprogramming that supports proliferation, invasion, therapeutic resistance, and adaptation to dynamic microenvironmental conditions. These alterations provide opportunities for metabolic imaging approaches that extend beyond conventional anatomical neuroimaging. Hyperpolarized magnetic resonance imaging (hpMRI) has emerged as a novel metabolic imaging platform capable of non-invasively visualizing real-time cellular metabolism through dynamic nuclear polarization of carbon-13-labeled substrates. By dramatically enhancing magnetic resonance signal intensity, hpMRI enables interrogation of enzyme-specific metabolic pathways and provides unique insight into tumor energetics, metabolic heterogeneity, and treatment response. The distinct contribution of this review is an updated, brain tumor-specific, clinically oriented framework that integrates recent human evidence with longitudinal metabolic phenotyping, emerging pathway-specific probes, acquisition standardization, multimodal validation, and the remaining barriers to clinical implementation. Particular emphasis is placed on hyperpolarized [1-^13^C]pyruvate, which has demonstrated feasibility and safety in patients with gliomas and has enabled assessment of glycolytic metabolism, oxidative phosphorylation, tumor recurrence, and longitudinal treatment response. Serial changes in lactate and bicarbonate flux may also reflect evolution toward more glycolytic, heterogeneous, and treatment-resistant tumor phenotypes, supporting the potential prognostic value of hpMRI before conventional radiographic progression becomes evident. We also review emerging applications involving α-ketoglutarate metabolism, redox biology, glutathione cycling, perfusion imaging, and molecular characterization of clinically relevant alterations including IDH1, TERT, and c-MYC-associated metabolic programs. In addition, we discuss recent advances in acquisition methods, image standardization, and multimodal integration with conventional MRI and positron emission tomography. Although several technical and logistical challenges remain, hpMRI is an investigational, radiation-free metabolic imaging modality with potential applications in diagnosis, molecular stratification, and treatment monitoring; however, substantial technical, regulatory, logistical, and economic barriers currently limit routine clinical use, molecular stratification, therapeutic monitoring, and precision medicine approaches in neuro-oncology. Continued clinical translation and development of novel metabolic probes may further expand its role in brain tumors and other neurological diseases.

## Simple Summary

Brain tumors undergo profound metabolic changes that drive tumor growth, treatment resistance, and disease progression. Conventional imaging primarily depicts anatomical features and often cannot identify biologic changes until structural progression has occurred. Hyperpolarized magnetic resonance imaging (hpMRI) is an emerging metabolic imaging technique that enables real-time, non-invasive visualization of cellular metabolism using hyperpolarized carbon-13-labeled substrates. Recent clinical studies have demonstrated the feasibility and safety of hpMRI in patients with gliomas and other brain tumors while highlighting its potential for assessing tumor metabolism, molecular characteristics, treatment response, and disease recurrence. This review summarizes current clinical applications of hpMRI in neuro-oncology, discusses emerging metabolic probes beyond pyruvate, and explores future opportunities for integrating metabolic imaging into precision medicine and image-guided therapeutic strategies.

## 1. Introduction

Brain tumors exhibit profound metabolic alterations that support proliferation, invasion, treatment resistance, and adaptation to hostile microenvironments [1,2,3]. Beyond the classical Warburg effect, gliomas and other brain malignancies demonstrate alterations across multiple interconnected metabolic pathways, including glycolysis, oxidative phosphorylation, glutamine metabolism, branched-chain amino acid utilization, redox homeostasis, ketone body metabolism, and the pentose phosphate pathway [3,4,5]. These shifts are often driven by tumor genotype and phenotype, including alterations in isocitrate dehydrogenase 1 (IDH1), telomerase reverse transcriptase (TERT), MYC proto-oncogene, bHLH transcription factor (c-MYC), and epidermal growth factor receptor (EGFR) signaling, and may evolve dynamically throughout disease progression and treatment [2,5,6]. Importantly, many of these metabolic adaptations contribute directly to radioresistance, chemotherapy resistance, immune evasion, and tumor recurrence [4,7]. The ability to characterize these metabolic changes non-invasively, longitudinally, and in real time has important implications for clinical neuro-oncology [8,9,10]. Knowledge of tumor metabolism could facilitate earlier diagnosis, improve molecular stratification, identify treatment resistance before structural progression becomes evident, and enable patient-specific therapeutic selection. Unlike conventional MRI, which primarily captures anatomy and contrast enhancement, metabolic imaging offers insight into the biologic state of tumors and their response to treatment.

Among the metabolites implicated in brain tumor biology, pyruvate occupies a central position in cellular energetics and serves as a key junction between glycolytic and oxidative metabolism [11,12]. In the healthy brain, pyruvate, a breakdown product of glucose, is transported across the blood–brain barrier (BBB) and cellular membranes by monocarboxylate transporters (MCTs), where it undergoes distinct cytosolic and mitochondrial fates [12,13]. Pyruvate may be converted to acetyl-CoA by the mitochondrial enzyme pyruvate dehydrogenase (PDH) to fuel oxidative phosphorylation or reduced to lactate by cytosolic lactate dehydrogenase (LDH) [12,14]. Under physiologic conditions, most ATP is generated through complete mitochondrial oxidation of glucose. However, during rapid proliferation and disease states such as cancer, cells often shift toward glycolysis to meet increased metabolic demand [2,15]. First described by Warburg in the early twentieth century, this metabolic adaptation enables cancer cells to generate energy at a substantially faster rate than oxidative respiration, even under conditions of adequate oxygen availability [15,16,17]. This preferential reliance on aerobic glycolysis, known as the Warburg effect, is thought to confer a selective advantage to cancer cells competing for limited nutrients and adapting to fluctuating tumor microenvironments.

Researchers and clinicians have increasingly sought to exploit these metabolic hallmarks for tumor detection and therapeutic monitoring. Several imaging modalities have been developed to characterize tumor metabolism in oncologic patients, including FDG-PET/CT and proton magnetic resonance spectroscopy (^1^H-MRS), both of which have improved tumor staging and assessment of brain bioenergetics. Nonetheless, important limitations remain. In the brain, the inherently high metabolic activity of normal parenchyma reduces the signal-to-noise ratio (SNR) of FDG-PET and ^1^H-MRS, complicating tumor detection and characterization [18,19]. Although advanced perfusion imaging assists in differentiating tumor recurrence from radiation necrosis, its ability to longitudinally assess treatment response remains limited [20]. Moreover, conventional imaging techniques often fail to detect therapeutic response or disease progression until morphologic or volumetric changes become apparent [8].

Hyperpolarized (HP) carbon-13 (^13^C) magnetic resonance imaging (MRI) has emerged as a promising metabolic imaging platform capable of interrogating cellular metabolism in a pathway-specific and real-time manner. By dramatically increasing the MR signal of ^13^C-labeled metabolites through dynamic nuclear polarization (DNP), hpMRI enables direct visualization of enzyme-specific metabolic reactions in vivo with signal enhancements approaching 10,000–100,000-fold [21]. This approach offers a safe, non-radioactive means of assessing malignant and benign brain tissue metabolism, with the potential to characterize tumor aggressiveness, monitor treatment response, evaluate recurrence, and identify molecular features relevant to prognosis and therapeutic resistance. Although much of the foundational work in hpMRI has been preclinical, recent human studies have demonstrated feasibility, safety, and early diagnostic utility in patients with gliomas and other brain malignancies.

**Figure 1:**
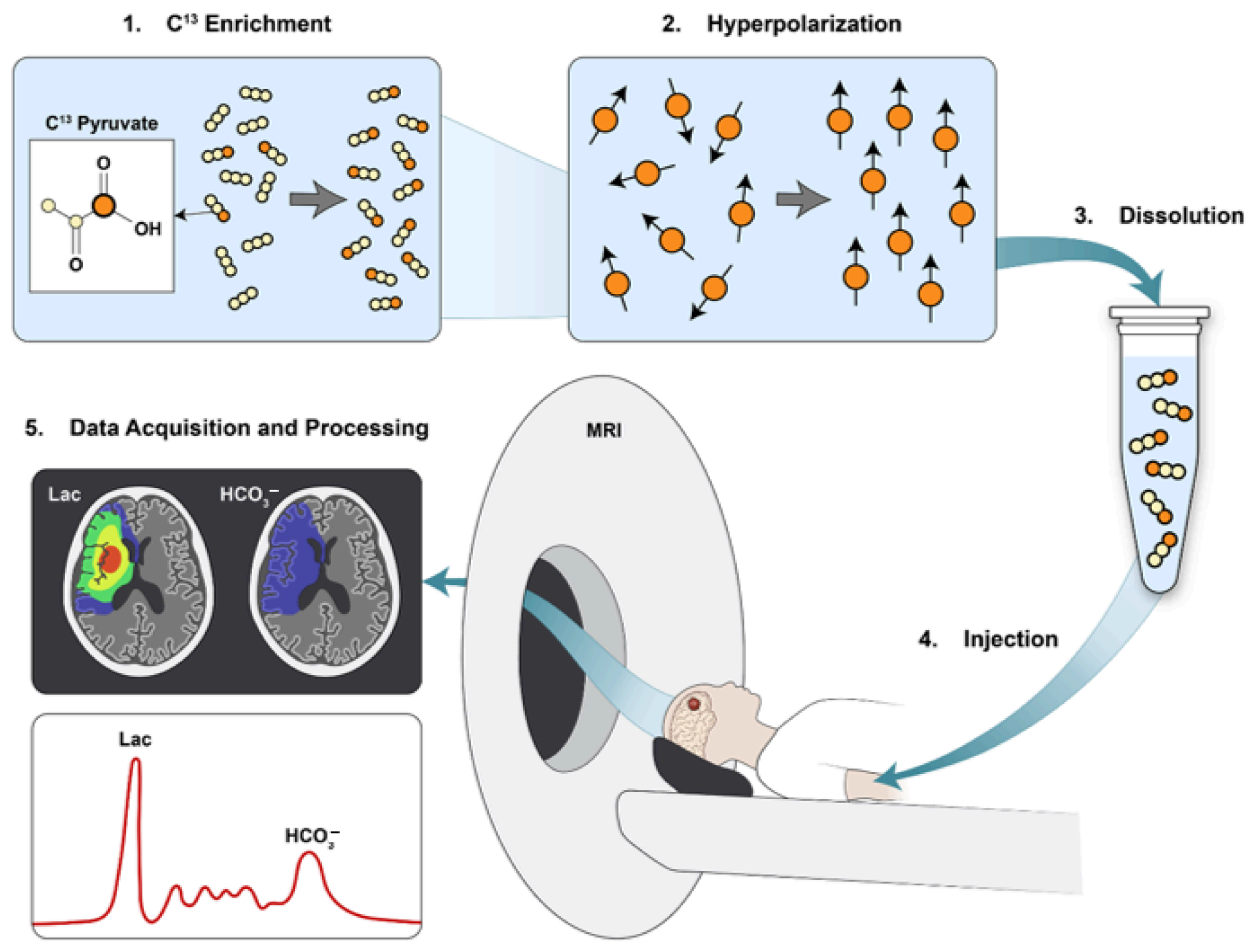
Hyperpolarized ^13^C MRI workflow from substrate preparation to metabolic imaging. Step 1: ^13^C enrichment. A highly concentrated solution of the molecule of interest is initially prepared (^13^C pyruvate shown in this figure). Step 2: Hyperpolarization. The sample is placed into a hyperpolarizer for ~1 h and is irradiated with microwaves at low temperatures (T 1–2 K). Step 3: Dissolution. The sample is at this point dissolved in a neutralizing solution to a pH of 7. At 37 °C, a final concentration of [1-^13^C]pyruvate of 5–100 mM is obtained. Step 4: Injection and MRI acquisition. The buffered solution is injected into the experimental subject and scanned with MRS over 10–15 s. Image acquisition is usually timed with the injection of the hyperpolarized tracer. Step 5: Data acquisition and processing. The acquired ^13^C signals are reconstructed as spectra and spatial metabolic maps co-registered with anatomical MRI. Representative schematic lactate and bicarbonate maps illustrate glycolytic and oxidative metabolic activity, respectively, while metabolite ratios and kinetic parameters can be used to quantify regional metabolic heterogeneity.

In identifying tumor metabolism and early recurrence, hpMRI may assist in molecular characterization, including determination of IDH status, TERT-associated metabolic activity, and c-MYC-related treatment resistance. Measurements of lactate, bicarbonate, and local pH may further improve assessment of necrosis, perfusion, and regional metabolic heterogeneity within tumors. As the technology evolves, hpMRI may become an important adjunct to conventional imaging by providing dynamic biologic information unavailable through anatomic imaging alone (Figure 1).

Here, we present a narrative review of the current clinical evidence supporting hpMRI in brain tumors, with emphasis on the metabolic pathways and biological processes interrogated using hyperpolarized ^13^C probes. Relevant studies are critically synthesized according to their methodological quality, consistency, clinical significance, and translational maturity. We discuss implications for tumor diagnosis, molecular stratification, prognostication, and treatment monitoring, while identifying unresolved questions and opportunities for integrating metabolic imaging into contemporary neuro-oncologic care.

## 2. Metabolic Pathways and Clinical Applications of hpMRI in Brain Tumors

The known metabolic alterations observed in brain tumors and their potential relevance to hyperpolarized magnetic resonance imaging (hpMRI) are summarized in Table 1. Brain malignancies exhibit dynamic rewiring across multiple metabolic pathways, including enhanced glycolysis, impaired oxidative phosphorylation, altered glutamine and branched-chain amino acid metabolism, dysregulated redox homeostasis, and increased pentose phosphate pathway activity [1,2,3,4,5,6,22]. Many of these shifts are closely linked to tumor aggressiveness, molecular subtype, therapeutic resistance, and recurrence, creating opportunities for pathway-specific metabolic imaging [1,2,3,4,5,6]. Although several candidate probes targeting these metabolic processes remain in preclinical development, pyruvate-based hpMRI has emerged as the first clinically translated approach for interrogating tumor metabolism in patients with brain tumors [9,10,23].

**Figure 2:**
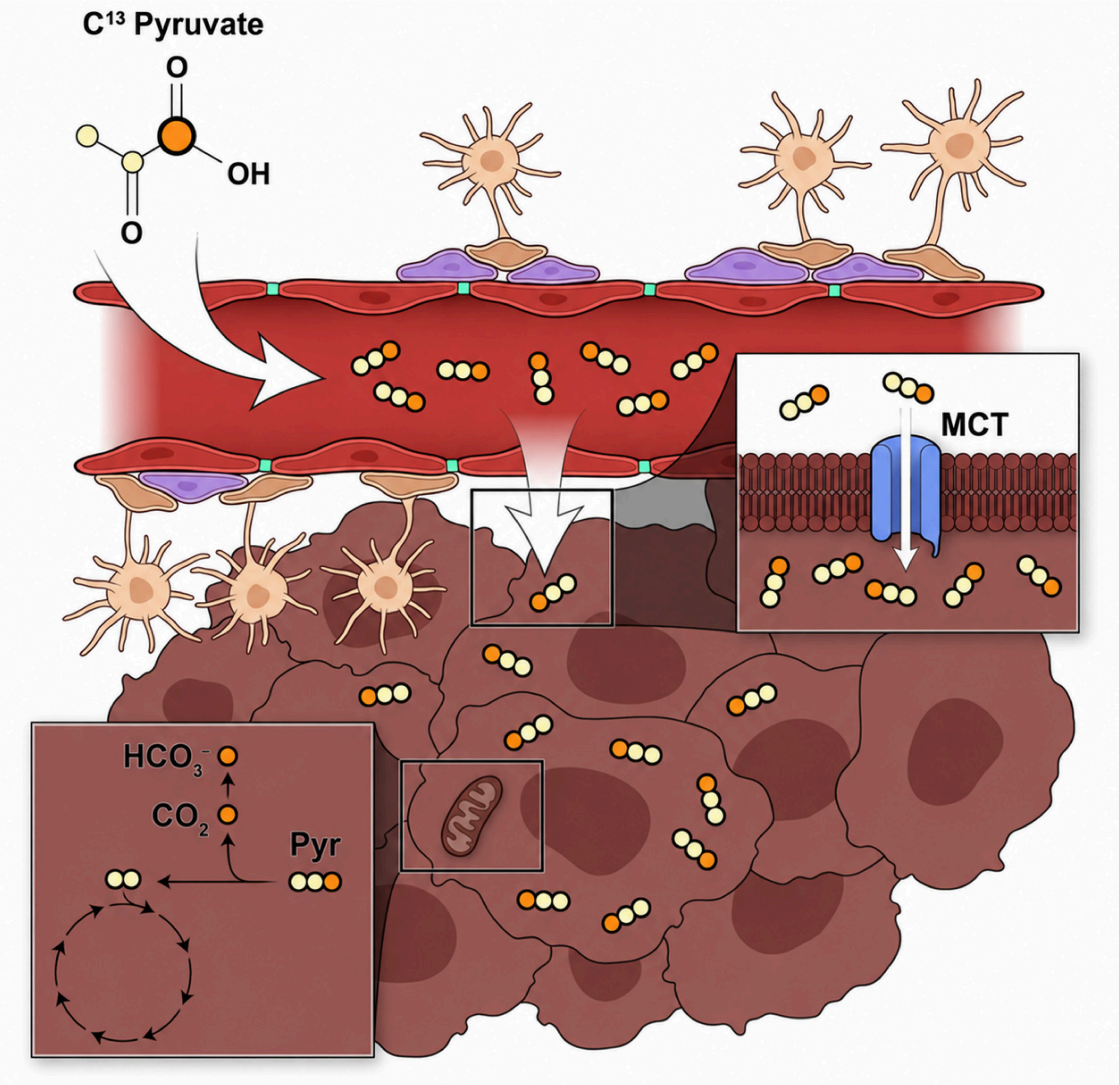
Schematic overview of ^13^C-hyperpolarized probe delivery and detection. Illustration of the in vivo transport and metabolic conversion of hyperpolarized ^13^C probes. Following intravenous administration, ^13^C-enriched tracers such as [1-^13^C]pyruvate cross the blood–brain barrier via monocarboxylate transporters (MCTs), diffuse into tumor cells, and are enzymatically converted into downstream metabolites (e.g., lactate, bicarbonate). These metabolic products are then visualized as distinct spectral peaks by hpMRI, providing real-time maps of glycolytic and oxidative fluxes.

In the clinical setting, hpMRI leverages carbon-13 (^13^C)-labeled metabolites to overcome the inherently low signal generated by endogenous ^13^C nuclei, which is limited by both low natural abundance and poor nuclear spin polarization [21]. Through dynamic nuclear polarization (DNP), electron polarization generated at low temperatures and high magnetic fields is transferred to ^13^C-enriched substrates, dramatically amplifying the magnetic resonance signal [21,40,43]. Following dissolution in a biologically compatible solution and intravenous administration, hyperpolarized probes enable real-time interrogation of metabolic reactions in vivo. This process increases ^13^C thermal equilibrium polarization from approximately 0.00025% to 30–40%, corresponding to an enhancement approaching 100,000-fold (Figure 2), thereby allowing high signal-to-noise ratio imaging of rapid enzymatic processes before polarization decay occurs [43]. Since hyperpolarized magnetization is non-renewable and decays according to the spin-lattice relaxation time (T1), typically within approximately 60–120 s after dissolution and injection, image acquisition must be completed rapidly to preserve metabolic signal. These temporal constraints have driven the development of ultrafast spatial-spectral acquisition strategies, including echo-planar imaging (EPI), spiral imaging, compressed sensing, and spectroscopic imaging methods that enable whole-brain metabolic mapping during the brief hyperpolarized imaging window.

In clinical practice, hpMRI data are typically displayed as spatial maps of the administered substrate and its downstream metabolites, co-registered with conventional anatomical MRI. Pyruvate maps primarily reflect substrate delivery and tissue uptake, lactate maps indicate LDH-mediated glycolytic labeling, and bicarbonate maps reflect PDH-mediated oxidative metabolism. Metabolite ratios and kinetic parameters, including k_PL and k_PB, may further quantify regional metabolic activity and longitudinal changes. These maps can reveal metabolic heterogeneity within enhancing and non-enhancing tumor regions, although interpretation must account for perfusion, blood–brain barrier permeability, acquisition timing, coil sensitivity, and the selected analysis model.

The earliest clinical applications of hpMRI in brain tumors focused on pyruvate metabolism because of the well-established glycolytic shift observed in malignant gliomas (Figure 2). Pyruvate occupies a central position in cellular energetics and can either be converted to lactate through lactate dehydrogenase (LDH), reflecting glycolytic metabolism, or enter mitochondrial oxidative metabolism through pyruvate dehydrogenase (PDH), producing bicarbonate as a downstream marker of oxidative phosphorylation. Progressive tumors typically demonstrate increased hyperpolarized [1-^13^C]pyruvate-to-[1-^13^C]lactate conversion and reduced [1-^13^C]pyruvate-to-[^13^C]bicarbonate flux relative to surrounding normal brain tissue, reflecting increased glycolysis and impaired oxidative metabolism [26,44].

More recently, the hpMRI metabolic repertoire has expanded beyond pyruvate to include probes targeting specific metabolic processes, including glycolysis (pyruvate), oxidative phosphorylation (bicarbonate), pentose phosphate pathway activity (gluconolactone), IDH-mutant metabolism (α-ketoglutarate), redox homeostasis (N-acetylcysteine), necrosis (fumarate), and perfusion (urea) (Table 1), including the pentose phosphate pathway, α-ketoglutarate metabolism, redox biology, glutathione cycling, and tumor perfusion [33,39,41,42]. These newer approaches offer the potential for non-invasive interrogation of clinically relevant molecular characteristics such as c-MYC, telomerase reverse transcriptase (TERT), and isocitrate dehydrogenase 1 (IDH1) status, as well as treatment resistance and oxidative stress responses. While most of these approaches remain in preclinical or early translational phases, pyruvate-based hpMRI has successfully transitioned to human studies and is increasingly being evaluated as an adjunct to conventional neuroimaging for treatment monitoring, recurrence detection, and metabolic stratification in glioma patients (Table 2). These novel techniques constitute a promising addition to current imaging approaches and open the field to further integration between diagnostic tools and image-guided molecular-driven surgery and radiation therapy.

Overall, the clinical evidence supporting hpMRI in brain tumors remains early-stage and methodologically heterogeneous. Most human investigations are single-center feasibility, pilot, or proof-of-principle studies involving small cohorts, variable tumor types and molecular subgroups, heterogeneous treatment exposures, and non-uniform imaging time points [42]. The principal demonstrated outcomes include safety, detection of pyruvate-to-lactate and pyruvate-to-bicarbonate conversion, characterization of regional metabolic heterogeneity, and preliminary associations with progression or treatment response. However, differences in radiofrequency hardware, acquisition sequences, injection timing, signal normalization, kinetic modeling, and reported metabolic endpoints limit direct comparison across studies. Most investigations were not designed to establish diagnostic accuracy, validated response thresholds, progression-free survival, or overall survival. Although the standardized protocol evaluated across 100 examinations in 42 patients provides encouraging evidence of within-center repeatability, independent multicenter and cross-platform reproducibility remain insufficiently established. Current evidence therefore supports the safety, feasibility, and biological relevance of hpMRI, while prospective multicenter studies with harmonized protocols and predefined outcome-linked endpoints are required before routine clinical implementation.

### 2.1. HP Pyruvate

Pyruvate, the product of glycolysis, has been a major focus of hpMRI studies in rodents and humans thanks to its position in the cellular energetic balance. Under normal conditions, pyruvate enters the mitochondrial tricarboxylic acid cycle via pyruvate dehydrogenase. On the other hand, in cancerous cells, this metabolite undergoes conversion into lactate via the cytosolic lactate dehydrogenase (LDH). Importantly, the hyperpolarized pyruvate-to-lactate signal reflects not only intracellular lactate dehydrogenase (LDH)-mediated exchange but also delivery of pyruvate to the tissue and transport across cellular membranes by monocarboxylate transporters (MCTs), particularly MCT1. Consequently, the apparent pyruvate-to-lactate conversion rate (kPL) represents a composite measure influenced by substrate delivery, transporter activity, intracellular metabolite pool size, and enzymatic exchange rather than LDH activity alone. This reaction can be exploited to label malignancies and follow their metabolic needs over time and in relation to treatment. Given its evolutionary importance and direct involvement in the metabolic switch observed in cancerous cells, the first animal studies in hpMRI have been conducted using [1-^13^C]pyruvate probes aimed at establishing and refining the ideal MRI sequences. Interpretation of brain hpMRI also depends critically on blood–brain barrier physiology and substrate delivery. Blood–brain barrier permeability varies across and within tumor types: contrast-enhancing glioblastoma and many brain metastases commonly contain regions of barrier disruption, whereas low-grade gliomas and infiltrative, non-enhancing margins may retain a relatively intact barrier. Hyperpolarized pyruvate delivery therefore may be spatially heterogeneous and does not necessarily correspond directly to tumor-cell density or metabolic activity. Transport across cellular membranes further depends on monocarboxylate transporters, particularly MCT1 and MCT4, whose expression may vary according to tumor subtype, hypoxia, and treatment state [12,13]. Apparent lactate and bicarbonate signals are therefore influenced by perfusion, blood–brain barrier permeability, MCT-mediated cellular uptake, intracellular substrate availability, and enzymatic conversion. In many settings, membrane transport rather than enzyme abundance may constitute the rate-limiting step governing the observed hyperpolarized signal [10,23,24,25,27]. A weak downstream signal may therefore reflect delivery limitation rather than low metabolic activity, whereas increased conversion may arise from enhanced delivery as well as altered metabolism. Co-registration with perfusion, permeability, and anatomical imaging and, where possible, kinetic modeling that separates substrate delivery from metabolic exchange is essential for distinguishing delivery-limited from metabolism-limited hpMRI signals.

Substrate pharmacokinetics further influence the hpMRI signal independently of tumor metabolism. Following intravenous administration, hyperpolarized substrates undergo systemic dilution, vascular dispersion, tissue uptake, possible peripheral metabolic conversion, renal clearance, and continuous T1-dependent polarization decay before and during brain imaging [21,39,40,41]. Consequently, measured pyruvate and downstream metabolite signals depend on the administered dose, injection rate, circulation time, cardiac output, perfusion, vascular volume, and dissolution-to-acquisition delay. Metabolite ratios may partially reduce delivery-related variability but do not completely separate substrate availability from enzymatic conversion, whereas kinetic models depend on assumptions regarding the vascular input function, exchange rates, relaxation, and tissue compartments [46,49]. Dynamic acquisition, co-polarized perfusion markers such as hyperpolarized urea, and integration with perfusion MRI may help distinguish delivery-related effects from true alterations in cellular metabolism [39].

HpMRI has emerged as a minimally invasive tool to detect early tumor recurrence and treatment response. This is achieved by measuring alterations in cellular metabolism and pyruvate concentrations before morphologic changes become evident on volumetric and contrast-enhanced MRI during early chemotherapeutic treatment.

After initial validation in several rodent models of glioma, non-human primates and human subjects have been enrolled to test the applicability of hpMRI to disease states and to treatment response. Given the importance of the glycolytic pathway for the energetic balance of astrocytic malignancies, particular emphasis has been placed on optimizing and safely translating hyperpolarized ^13^C-based imaging in these patients. This technique may in fact support and assist classic MRI spectroscopy and perfusion in assessing early treatment response and local progression [55,56]. While safety and scalability have been at the center of most studies, current evidence supports the continued clinical evaluation of hpMRI as an adjunct to conventional imaging; however, routine implementation remains limited by specialized hardware, operational complexity, cost, and insufficient multicenter validation.

Grist et al. intravenously injected hyperpolarized [1-^13^C]pyruvate in the brain of healthy human volunteers for the first time in 2019, showing that both lactate and bicarbonate can be probed in vivo, despite the presence of an intact BBB. Unsurprisingly, [1-^13^C]pyruvate, [1-^13^C]lactate, and [^13^C]bicarbonate concentrations were higher in gray matter compared to white matter tracts [27]. In another study, Park et al. turned to glioma patients to test new hpMRI strategies to probe cellular metabolism in vivo. While all three metabolites were visible in healthy brain tissue, only pyruvate and lactate were detected in the tumor. Further, the use of [1-^13^C]pyruvate proved to be safe in human subjects [23]. Miloushev et al. showed evidence of hpMRI signal in both tumor and healthy brain in a cohort of recurrent and newly diagnosed glioma patients. Results correlated with MRI, MRI DCE perfusion, and FDG PET/CT, suggesting that pyruvate-to-lactate conversion may be a quantitative metabolic biomarker useful for assessing tumor response [42]. Autry et al. compared healthy volunteers to infiltrating glioma patients during treatment using [1-^13^C]pyruvate hpMRI echo-planar imaging. Scans were performed at clinical endpoints that overlapped with treatments (XRT, TMZ and bevacizumab). Apparent rate constants for [1-^13^C]pyruvate conversion to [1-^13^C]lactate (kPL) and [^13^C]bicarbonate (kPB) were found to be globally elevated after bevacizumab use. Interestingly, in three patients with disease progression, neoplastic lesions showed elevated kPL compared to the normal-appearing white matter despite a lack of Gadolinium enhancement on T1 sequences [10].

In a recent study, Zaccagna et al. investigated treatment-naïve glioblastoma patients and compared tumoral tissue to normal brain parenchyma metabolism. A lower bicarbonate-to-pyruvate ratio was measured in cancerous regions, while the lactate-to-pyruvate ratio appeared unchanged between healthy and diseased cells. A strong correlation between tumor lactate and bicarbonate signals was noticed. Interestingly, this group reported that while lactate levels tend to vary significantly within the same malignancy and between different tumors, bicarbonate labeling appears to be stably decreased in cancerous lesions [24]. Autry et al. compared treatment-naïve and post-treatment glioblastoma and high-grade glioma patients with [1-^13^C]pyruvate tracer. Glioblastoma displayed lower perfusion %-recovery and rate constants for [1-^13^C]pyruvate-to-[1-^13^C]lactate conversion (kPL), higher 1H-lactate and [1-^13^C]pyruvate levels compared to IDH-mutant astrocytomas. Higher cerebral blood volume (nCBV), kPL, and [1-^13^C]pyruvate-to-[1-^13^C]lactate were observed in glioblastoma after treatment [25].

Collectively, these findings suggest that hpMRI may characterize the biological evolution of glioblastoma rather than merely detect tumor presence. Increasing pyruvate-to-lactate conversion, reduced bicarbonate production, and greater spatial metabolic heterogeneity may reflect progression toward a more glycolytic, aggressive, and treatment-adapted phenotype. Importantly, elevated kPL in progressing non-enhancing lesions suggests that metabolic evolution may precede or extend beyond conventional contrast-enhancing progression. Serial within-patient hpMRI may therefore provide greater prognostic information than isolated measurements, although validation in larger outcome-linked cohorts remains necessary.

Although [1-^13^C]pyruvate remains the most clinically mature hyperpolarized substrate, a growing range of alternative probes is being developed to interrogate complementary features of brain tumor biology. These substrates differ substantially in biological specificity, polarization lifetime, transport across the blood–brain barrier, metabolic conversion rate, signal strength, and readiness for human translation. Hyperpolarized α-ketoglutarate exploits mutant isocitrate dehydrogenase (IDH)-mediated conversion to 2-hydroxyglutarate, providing a potential imaging biomarker for molecular classification of diffuse gliomas and pharmacodynamic monitoring of IDH-targeted therapies. Hyperpolarized glutamine-related probes may assess glutamine dependence and anaplerotic metabolism, but their interpretation is complicated by rapid metabolism, multiple downstream products, and spectral overlap. Hyperpolarized [1,4-^13^C2]fumarate measures conversion to malate by fumarate hydratase following loss of cell membrane integrity. Because intracellular fumarase becomes accessible only after membrane disruption, this probe serves primarily as an imaging biomarker of treatment-induced necrosis rather than baseline tumor metabolism. Hyperpolarized δ-[1-^13^C]gluconolactone targets glucose-6-phosphate dehydrogenase and pentose phosphate pathway activity, offering a potential readout of TERT-associated metabolism and redox adaptation. Hyperpolarized N-acetylcysteine probes glutathione-dependent thiol redox chemistry and may help evaluate oxidative stress and ferroptosis-related vulnerabilities. Acetoacetate-based probes may characterize ketone body utilization and metabolic flexibility, whereas hyperpolarized urea provides a largely non-metabolized marker of perfusion and vascular delivery that can be co-polarized with metabolic substrates to separate substrate delivery from intracellular conversion. Emerging 5-aminolevulinic acid-based approaches seek to interrogate tumor-selective heme biosynthesis and may ultimately support theranostic applications involving fluorescence, photodynamic therapy, or sonodynamic therapy. However, most probes beyond pyruvate remain preclinical or early translational. Common barriers include limited brain delivery, weak or rapidly decaying product signals, spectral overlap, probe-specific toxicity and manufacturing requirements, regulatory approval, and the absence of validated clinical thresholds. Their most likely near-term role is therefore complementary: molecular subtype identification, pharmacodynamic monitoring, treatment-response assessment, redox phenotyping, and separation of perfusion from metabolic flux.

#### 2.1.1. HP Pyruvate in Pediatric Brainstem Gliomas

Diffuse intrinsic pontine gliomas (DIPGs) are an aggressive subtype of astrocytic tumors that, despite variable histology, almost universally result in poor survival outcomes [57].

Tumor grade and histology, as well as the limited surgical and therapeutic options available, are still a challenge in these malignancies, considering the need for tissue analysis and the surgical risks that a brainstem biopsy poses. Further, current imaging techniques do not allow for accurate estimates of functional changes and treatment response and cannot stratify disease aggressiveness [58,59,60,61]. Metabolic imaging would therefore introduce a new approach to study disease progression, response to chemoradiation, and specific tumor mutations in a non-invasive way. To this day, only one study has attempted to test hpMRI imaging in this cohort.

Autry et al. first introduced pyruvate-based hpMRI in DIPG patients, imaging several pediatric brain tumor patients with hp-[1-^13^C]pyruvate. Two dose levels were tested in six participants, and no side effects or toxicities were reported. Conversion to [1-^13^C]lactate and [^13^C]bicarbonate in the brain was noted in all subjects, demonstrating the feasibility and safety of this approach in a challenging oncologic population [48].

#### 2.1.2. HP Pyruvate in Brain Metastases

Beyond gliomas, hp-13C biomarkers are starting to predict therapy outcomes in other brain tumors. In patients with brain metastases undergoing stereotactic radiosurgery (SRS), Cappelletto et al. showed that the pretreatment lactate-to-bicarbonate signal ratio derived from hp-[1-^13^C]pyruvate imaging predicts post-SRS response [50]. A higher Lac:HCO3− ratio reflecting a more glycolytic and less oxidative phenotype was associated with poorer local control, whereas lower ratios predicted favorable response. These data position hp-^13^C as a non-invasive metabolic stratifier for radiation sensitivity, potentially guiding dose adaptation or adjunct systemic therapy selection in metastatic brain disease.

### 2.2. Novel Coils and Acquisition Methods

Despite the growing field of hpMRI, several technical aspects still need to be developed to optimize the hardware resolution, SNR, and coverage. These improvements are generally aimed at reducing the invasiveness of the technique and ameliorating its clinical applicability and sensitivity/specificity. In an effort to optimize the performance of current hpMRI technology, Autry et al. compared 8- and 32-channel receive coils in a single-patient study [47]. While the 8-channel receiving array showed SNR benefits along the brain periphery, the 32-channel array exhibited better coverage and a more uniform profile across different regions of the brain.

Several other groups attempted to improve the sensitivity and signal-to-noise (SNR) ratio of hpMRI in cancerous lesions. This has been done mainly via optimization of MRI sequences and the post-processing process. Vaziri et al. tested two HOSVD denoising techniques, tensor rank truncation-image enhancement (TRI) and global–local HOSVD (GL-HOSVD) voxel-wise evaluation, in glioma patients.

Higher metabolite SNR and regional signal coverage were reported, and the number of voxels showing within-tolerance kPL modeling error relative to the total voxels almost doubled [54]. Crane et al. also provided several examples of 2D and 3D sequences applied to the human brain to support inter-institutional use and standardization [62]. Gordon et al. tested a symmetric EPI rapid sequence with 3s resolution in brain tumor patients and confirmed the feasibility of this technique by achieving whole organ coverage and detection of all three metabolites in the brain parenchyma [45].

Mammoli et al. tried to assess the rate of conversion to lactate (kPL) and bicarbonate (kPB) with echo-planar imaging and explored different models for data fitting. Input-less models appeared to best agree with the data [46].

Clinical hpMRI requires an MRI system equipped for multinuclear or X-nucleus imaging, including a dedicated ^13^C transmit–receive chain and either dedicated ^13^C or dual-tuned 1H/^13^C radiofrequency coils. These capabilities are generally optional rather than standard on clinical scanners and remain concentrated in specialized academic centers. Pulse sequences must also be optimized to balance rapid acquisition, spectral separation, spatial coverage, signal-to-noise ratio, and preservation of the non-renewable hyperpolarized magnetization. Consequently, the spatial resolution of hpMRI remains lower than that of conventional proton MRI, while temporal resolution is constrained by the short T1 relaxation time and narrow post-injection imaging window. Quantification further depends on model assumptions regarding substrate delivery, exchange kinetics, relaxation, flip-angle calibration, input functions, and compartmental behavior. Metabolite ratios and fitted rate constants such as kPL and kPB are therefore not interchangeable and may vary according to coil sensitivity, perfusion, blood–brain barrier permeability, acquisition timing, and the selected kinetic model. These hardware, sequence, and modeling constraints currently limit inter-scanner comparability and represent major barriers to routine implementation outside experienced centers [21,40,41,45,46,47,49,54,62]. Hyperpolarization represents an artificially generated, non-equilibrium magnetization that decays approximately exponentially according to the substrate-specific T1 relaxation time and is irreversibly consumed by radiofrequency excitation [21,40,43]. Imaging must therefore occur within a narrow post-dissolution window, requiring close proximity between the polarizer and MRI scanner, rapid probe quality control and transfer, and tight synchronization of dissolution, injection, and acquisition. Once polarization has decayed, the signal cannot be recovered without preparing and administering a newly hyperpolarized dose, limiting repeated acquisitions from a single injection. Current mitigation strategies include rapid dissolution and automated injection workflows, standardized timing procedures, fast echo-planar and spectrally selective sequences, variable-flip-angle schemes that conserve magnetization, and development of probes with longer T1 relaxation times [41,45,49,62]. Nevertheless, polarization decay remains a fundamental constraint on scan duration, spatial coverage, signal-to-noise ratio, and multicenter workflow reproducibility. Standardization of human hp-^13^C workflows is accelerating. Autry et al. reported an advanced clinical protocol for glioma patients that harmonizes coil selection, polarization/dissolution parameters, timing of injection and acquisition, whole-brain EPI-based coverage, and kinetic modeling of k_PL and k_PB across scanners and visits [49]. The protocol formalizes QA steps (polarization yield, pyruvate concentration/pH/temperature), prescribes scheduling relative to chemo-radiation and anti-angiogenic therapy, and details co-registration with 1H maps (perfusion/DCE, CNI). Adoption of such standardized pipelines should improve multi-site reproducibility, facilitate longitudinal response assessment, and enable meta-analytic pooling of hp-^13^C endpoints in future trials. For reproducible clinical implementation, acquisition protocols should consistently report polarization yield, probe concentration, pH and temperature, administered dose, injection rate, dissolution-to-acquisition delay, radiofrequency coil configuration, flip-angle scheme, and spatial and temporal resolution. Reconstruction pipelines should document coil combination, denoising, motion correction, spectral separation, and co-registration with anatomical MRI. Quantification methods should distinguish metabolite ratios from kinetic parameters such as kPL and kPB and should report normalization procedures, model assumptions, uncertainty estimates, and voxel-level quality-control criteria. Standardized phantoms, scanner calibration, test–retest imaging, and harmonized quality-assurance procedures will be necessary to evaluate inter-scanner and inter-institutional variability.

### 2.3. Orthogonal Validation with Metabolic PET and Spatial Heterogeneity

The spatial specificity of hp-^13^C readouts in the human brain is supported by orthogonal validation against metabolic PET. Blazey et al. found region-dependent correspondence and biologically significant divergences when they compared metabolic PET distributions with hp-[1-^13^C]pyruvate MRI maps (pyruvate, lactate, and bicarbonate) [51]. While PET focused on tracer uptake/retention kinetics collectively, revealing intratumoral metabolic heterogeneity not captured by any single modality, hp-^13^C focused on rapid pathway flux (e.g., lactate labeling and bicarbonate as a surrogate of PDH flux). These results support the use of integrated hp-^13^C/18F-PET paradigms in studies aiming to monitor therapy and obtain thorough metabolic phenotyping.

### 2.4. Comparison with Conventional and Advanced Neuroimaging

Conventional and advanced neuroimaging modalities provide complementary but biologically distinct information in brain tumors. Contrast-enhanced MRI remains the standard for anatomical localization, surgical planning, and longitudinal response assessment, but enhancement primarily reflects blood–brain barrier disruption and may not reliably distinguish viable tumors from pseudoprogression, radiation injury, inflammation, or treatment-related vascular changes. Diffusion-weighted imaging provides an indirect measure of cellularity through the apparent diffusion coefficient, whereas perfusion MRI evaluates vascularity and blood flow using parameters such as relative cerebral blood volume, cerebral blood flow, and contrast-transfer coefficients. Proton magnetic resonance spectroscopy measures steady-state metabolite pools, including choline, N-acetylaspartate, creatine, lactate, and 2-hydroxyglutarate, while PET evaluates radiotracer uptake and retention over several minutes. In contrast, hpMRI measures the rapid enzymatic conversion of an administered ^13^C-labeled substrate into downstream metabolites over seconds (Table 3). Therefore, hpMRI should be considered complementary to, rather than a replacement for, established anatomical, physiological, and molecular imaging approaches.

These results should not be interpreted as direct evidence that one modality is superior to another, because the reported estimates were derived from different populations, endpoints, reference standards, and acquisition protocols. In particular, adequately powered head-to-head studies comparing the sensitivity, specificity, and incremental clinical value of hpMRI with conventional MRI, perfusion, diffusion, spectroscopy, and PET have yet to be performed. The principal potential advantage of hpMRI is therefore its unique ability to measure pathway-specific metabolic flux with high temporal resolution, rather than established superiority in diagnostic accuracy.

## 3. Discussion

Hyperpolarized MRI, a new non-invasive, safe, real-time and non-radioactive MRS technique for imaging metabolic pathways in vivo, has recently gained popularity after several rodent studies and the first promising human trials. Several studies using hyperpolarized [1-^13^C]pyruvate to interrogate the glycolytic cycle have confirmed early changes in HGG and DIPG. Further, these changes can be tracked and followed over time.

### 3.1. HpMRI for Early Diagnosis and Stratification of HGG

Several pathways can be interrogated to visualize early carcinogenesis and tumor growth with hpMRI. Specifically, the Warburg effect has been extensively used in both HGG and DIPG with pyruvate-based probes. This pathway, despite being frequently upregulated in malignant cells, might be challenging to image in organs with high baseline metabolism, such as the brain. Further, inflammatory reactions linked with the primary immune response or immunostimulatory treatments may confound the results and lead to an overestimate of the tumor size. To obviate this limitation, other metabolic cascades have been interrogated in vivo and will likely be translated to primate and human studies in the future. Alterations in the branched-chain amino acids and leucine metabolism [29,30,37], the pentose phosphate [28,31,32] and acetoacetate/β-hydroxybutyrate pathways [6,34], and the glutamate/glutamine cycle [35,36,67] have been suggested as markers of glioma genesis, based on their dysregulation seen in these malignancies.

### 3.2. Longitudinal Tumor Evolution, Prognostication, and Treatment Response

Glioblastoma evolves continuously under selective pressures imposed by hypoxia, nutrient limitation, surgery, radiotherapy, chemotherapy, and anti-angiogenic treatment. These pressures may promote cellular states characterized by increased glycolysis, metabolic flexibility, invasiveness, and treatment resistance. Since hpMRI measures pathway-specific metabolic activity, serial imaging could provide a non-invasive record of this biological evolution. Persistent or increasing kPL, a rising lactate-to-bicarbonate ratio, reduced bicarbonate labeling, or expansion of metabolic abnormalities into non-enhancing tissue may indicate residual viable disease or emergence of a more aggressive phenotype. Available clinical studies support this concept by demonstrating elevated kPL in progressing glioma, heterogeneous lactate labeling and reduced bicarbonate production in glioblastoma, and an association between higher lactate-to-bicarbonate ratios and poorer local treatment response. Nevertheless, the prognostic value of hpMRI in glioblastoma remains investigational and requires prospective validation against progression-free survival, overall survival, and spatial patterns of recurrence.

Finally, while this review revolves around the use of hpMRI in brain tumors, where a detailed description of the anatomy is vital for surgical and practical reasons, the same level of morphologic accuracy is not necessarily needed in other organs. Specifically, malignancies of the lymphoid system are diffuse forms of cancer that usually do not benefit from the use of advanced imaging techniques. Nonetheless, hpMRI could represent an interesting adjunct to molecular tests in assessing treatment response and in testing new mutations such as IDH-1. Limiting a scan to hpMRI, without the anatomic and volumetric correlate, would, in fact, greatly simplify the infrastructure needed and overall costs, making this technology a readily available technique to sample and test in vivo treatment response and overall disease burden and evolution.

### 3.3. Non-Invasive Analysis of IDH-1 Status with hpMRI

Alpha-ketoglutarate is a tricarboxylic acid-cycle intermediate that also serves as a substrate for transaminases and alpha-ketoglutarate-dependent dioxygenases involved in DNA and histone demethylation. Wild-type IDH1 converts isocitrate to alpha-ketoglutarate while generating NADPH, thereby supporting cellular redox homeostasis and biosynthesis. Mutant IDH1 acquires a neomorphic enzymatic activity that consumes alpha-ketoglutarate and NADPH to produce D-2-hydroxyglutarate. Accumulated 2-hydroxyglutarate competitively inhibits alpha-ketoglutarate-dependent dioxygenases, producing widespread epigenetic remodeling, impaired cellular differentiation, and the glioma CpG island methylator phenotype.

Hyperpolarized [1-^13^C]alpha-ketoglutarate provides a pathway-specific approach for interrogating this altered enzymatic activity. In IDH1-mutant glioma models, the administered probe is converted to hyperpolarized [1-^13^C]2-hydroxyglutarate, whereas this product is not detected in otherwise comparable IDH1-wild-type tumors. Alpha-ketoglutarate may also undergo transamination to glutamate, providing information regarding normal alpha-ketoglutarate utilization and potentially serving as a complementary metabolic endpoint. These measurements could support non-invasive molecular classification and pharmacodynamic monitoring of mutant-IDH inhibitors by demonstrating reduced 2-hydroxyglutarate production. However, the evidence remains predominantly preclinical or early translational, and sensitivity, specificity, and reproducibility in human glioma require prospective validation. Collectively, coupling hp-^13^C pyruvate metrics to microenvironmental pH [67], adopting standardized acquisition/analysis pipelines in glioma [49], extending prognostic utility to brain metastases treated with SRS [50], and cross-validating spatial patterns with metabolic PET [51] delineate the next phase of clinical translation. These advances suggest hp-^13^C can move from feasibility to decision-making, where metabolic phenotypes inform patient selection, dose adaptation, and early switch criteria across primary and metastatic brain tumors.

Beyond IDH1, hpMRI may provide metabolic signatures of additional molecularly defined glioma subtypes. TERT-associated upregulation of glucose-6-phosphate dehydrogenase and pentose phosphate pathway activity may be interrogated using hyperpolarized δ-[1-^13^C]gluconolactone, whereas c-MYC-driven glycolytic and glutamine-dependent programs may be reflected by increased pyruvate-to-lactate conversion and altered glutamine metabolism. Branched-chain amino acid tracers may similarly provide information regarding BCAT1-associated, IDH-wild-type aggressive phenotypes. These pathway-specific readouts could support non-invasive molecular stratification, selection of metabolism-directed therapies, and pharmacodynamic monitoring. However, most applications beyond pyruvate remain preclinical and require validation before hpMRI can reliably distinguish molecular subtypes in clinical practice.

### 3.4. Redox Imaging and Leveraging Metabolic Vulnerabilities for Novel Therapy

An emerging application of hyperpolarized MRI (hpMRI) in neuro-oncology is the interrogation of tumor redox state, a critical determinant of glioblastoma (GBM) survival, treatment resistance, and susceptibility to oxidative stress-based therapies. While current clinical hpMRI studies have largely focused on glycolytic flux through pyruvate-to-lactate conversion, expanding this platform to directly probe redox metabolism may provide complementary insight into tumor biology and therapeutic response. Recent studies have identified cysteine metabolism as a key metabolic vulnerability in GBM, where the disruption of glutathione (GSH)-dependent antioxidant systems induces oxidative stress and ferroptotic cell death [67,68]. In this context, cyst(e)inase, a human enzyme that depletes cysteine and cystine, has demonstrated anti-tumor efficacy in glioma stem cell models and orthotopic tumors by inducing profound redox imbalance characterized by GSH depletion, reactive oxygen species accumulation, lipid peroxidation, and prolonged survival, including in temozolomide-resistant models [69]. The glutathione system links cysteine availability, NADPH production, oxidative-stress buffering, and ferroptosis resistance. Reduced glutathione is synthesized from glutamate, cysteine, and glycine and is consumed by glutathione peroxidases, including GPX4, to reduce hydrogen peroxide and lipid hydroperoxides. During this reaction, reduced glutathione is oxidized to glutathione disulfide, which is subsequently converted back to reduced glutathione by glutathione reductase using NADPH. Glioblastoma cells can reinforce this cycle through increased cystine uptake, cysteine utilization, pentose phosphate pathway activity, and NADPH generation, thereby limiting lipid peroxidation and supporting survival during hypoxia, radiotherapy, chemotherapy, and other oxidative stresses. Disruption of cysteine availability or GPX4-dependent lipid-peroxide detoxification can overwhelm this antioxidant system and promote ferroptotic cell death.

Hyperpolarized redox probes are intended to measure dynamic chemical reactions within this system rather than simply quantify total glutathione concentration. Hyperpolarized [1-^13^C]N-acetylcysteine can undergo redox-dependent thiol–disulfide exchange, including the formation of an N-acetylcysteine–glutathione mixed disulfide with a spectrally distinguishable resonance. The relative substrate and product signals may therefore provide a real-time indicator of tissue redox chemistry and glutathione-dependent buffering. Clinically, such imaging could identify redox-dependent tumors, map intratumoral oxidative-stress heterogeneity, and provide an early pharmacodynamic readout of therapies targeting cysteine metabolism or ferroptosis resistance. However, this probe remains preclinical, and its signal may also be influenced by delivery, tissue uptake, probe concentration, and competing thiol reactions.

Given that cyst(e)inase exerts its therapeutic effects through disruption of redox homeostasis, redox-sensitive hpMRI probes may provide a non-invasive means to monitor treatment response in vivo. Hyperpolarized [1-^13^C]N-acetylcysteine (HP-[1-^13^C]NAC) has been evaluated in preclinical proof-of-concept studies as a potential probe of intracellular thiol buffering and glutathione metabolism; it has not yet been validated in patients with brain tumors. It directly participates in intracellular thiol buffering and glutathione metabolism [69], enabling pathway-specific assessment of oxidative stress and antioxidant capacity. Following hyperpolarization and intravenous administration, HP-NAC could be integrated into established hpMRI workflows to quantify treatment-induced changes in redox balance through alterations in NAC-derived metabolic signals. Serial imaging before and after cyst(e)inase therapy, coupled with co-registration to conventional 1H MRI and perfusion imaging, may permit spatial mapping of redox heterogeneity and early detection of therapeutic response before volumetric changes occur. More broadly, integration of redox-sensitive probes into hpMRI expands the modality beyond energy metabolism to include oxidative stress biology, potentially enabling patient stratification and monitoring of therapies targeting metabolic vulnerabilities in brain tumors. Accordingly, proposed applications for patient stratification or treatment monitoring remain hypothetical and require validation of brain delivery, signal specificity, safety, reproducibility, and clinical utility.

### 3.5. 5-Aminolevulinic Acid Metabolism and Emerging Theranostic Applications of hpMRI

An additional metabolic pathway of growing interest in neuro-oncology involves the metabolism of 5-aminolevulinic acid (5-ALA), a naturally occurring precursor in heme biosynthesis that is selectively upregulated in glioblastoma and forms the basis of fluorescence-guided surgery (FGS) [70,71,72]]. Following systemic administration, 5-ALA is metabolized into protoporphyrin IX (PpIX), a fluorescent chromophore that preferentially accumulates within malignant glioma cells because of tumor-specific enzymatic and transporter alterations. Clinically, 5-ALA-guided resection has improved intraoperative tumor visualization and extent of resection and has become an established adjunct in high-grade glioma surgery [72,73]. These observations suggest that altered 5-ALA metabolism represents a clinically validated metabolic vulnerability that may be amenable to hyperpolarized imaging.

Extending principles established through pyruvate-based hpMRI, hyperpolarized carbon-^13^-labeled 5-ALA remains a conceptual and preclinical imaging strategy. Although it has been proposed as a pathway-specific approach for interrogating tumor-selective heme biosynthesis, no clinical hpMRI studies have yet demonstrated its feasibility, safety, or diagnostic performance in patients. In principle, hyperpolarized [1-^13^C]5-ALA could enable non-invasive visualization of metabolic flux through the 5-ALA-to-PpIX pathway, potentially allowing longitudinal assessment of tumor metabolism, treatment response, and regional heterogeneity beyond what is possible with intraoperative fluorescence alone [73,74]. Importantly, because PpIX also functions as a sensitizer for photodynamic and sonodynamic therapies, integration of 5-ALA-based hpMRI may ultimately support a theranostic framework in which tumor-specific metabolism is simultaneously leveraged for imaging, molecular characterization, and targeted treatment [75,76]. While this application remains preclinical and has not yet undergone human translation, it represents a compelling future direction for metabolite-specific imaging in glioblastoma.

### 3.6. Current Limitations and Barriers to Clinical Translation

Despite its potential for non-invasive metabolic phenotyping, several limitations currently restrict the broader clinical implementation of hpMRI. First, the technique requires access to specialized dynamic nuclear polarization equipment, dedicated ^13^C radiofrequency coils, compatible acquisition sequences, sterile probe-preparation and quality-control infrastructure, and personnel with expertise in metabolic imaging, MR physics, pharmacy, and image analysis. These requirements limit availability to a relatively small number of specialized centers and contribute to substantial capital, maintenance, staffing, and per-examination costs.

A major technical constraint is the short lifetime of the hyperpolarized signal. Following dissolution, polarization decays rapidly through T1 relaxation and is further depleted by radiofrequency excitation, leaving a narrow time window for probe quality assessment, administration, and image acquisition. Variations in polarization level, probe concentration, pH, temperature, dissolution-to-injection delay, injection rate, acquisition timing, coil sensitivity, pulse sequence, and reconstruction approach may therefore influence the measured metabolic signal. In addition, lactate, bicarbonate, and kinetic-rate measurements are affected not only by intracellular metabolism but also by substrate delivery, tumor perfusion, blood–brain barrier permeability, monocarboxylate transporter expression, and the assumptions of the selected kinetic model.

These factors also create challenges for reproducibility and multicenter implementation. Although recent standardized protocols have demonstrated encouraging within-center repeatability, acquisition hardware, calibration procedures, signal normalization, kinetic modeling, and reported metabolic endpoints remain heterogeneous across institutions. Robust multicenter translation will require harmonized probe-production procedures, scanner calibration, acquisition timing, quality-assurance criteria, reconstruction methods, and analysis pipelines, together with predefined clinically meaningful thresholds.

Safety evidence is currently strongest for hyperpolarized [1-^13^C]pyruvate and is based on relatively small early-phase clinical cohorts demonstrating acceptable short-term tolerability. Clinical administration nevertheless requires strict control of substrate dose, solution concentration, osmolality, pH, temperature, sterility, endotoxin levels, and residual free radicals following dissolution. Injection rate and cardiovascular or metabolic effects must also be monitored, particularly with repeated administrations, for which cumulative safety data remain limited. Importantly, safety findings for pyruvate cannot be extrapolated to other hyperpolarized substrates, because each probe and its downstream metabolites may have distinct toxicological, pharmacokinetic, and dose-related properties. Future probes will therefore require independent preclinical toxicology, manufacturing validation, regulatory review, and prospective human safety assessment. Finally, hyperpolarized ^13^C probes remain investigational imaging agents. Clinical administration requires regulatory authorization, controlled manufacturing, sterility testing, and predefined release criteria. Each probe beyond [1-^13^C]pyruvate will require separate toxicological, manufacturing, and regulatory evaluation, potentially slowing clinical translation. Accordingly, broader adoption will depend on multicenter validation, demonstrated clinical benefit beyond conventional MRI and PET, cost-effectiveness analyses, and evidence that hpMRI-derived measurements can reproducibly influence patient management and outcomes. Key research priorities include prospective multicenter trials using harmonized acquisition, reconstruction, and quantification protocols; cross-vendor and cross-scanner reproducibility studies; and validation against spatially matched histopathology, molecular data, progression-free survival, and overall survival. Integration with artificial intelligence and radiomics may enable automated segmentation, denoising, metabolic-feature extraction, and prediction of treatment response, but such models will require transparent reporting and external validation. Combining hpMRI with genomic, transcriptomic, proteomic, and metabolomic data may further connect spatial metabolic phenotypes with tumor cell states and therapeutic vulnerabilities. Ultimately, prospective trials must demonstrate that hpMRI provides incremental clinical utility, improves treatment decisions, and is cost-effective relative to established imaging approaches.

Within existing neuro-oncology workflows, hpMRI would most likely serve as a complementary problem-solving tool rather than replace conventional MRI. Initial use may be most appropriate for selected patients with high-grade glioma in whom conventional imaging is equivocal, early treatment response is uncertain, or metabolic characterization could influence biopsy targeting, radiation planning, or therapy selection. Serial hpMRI could be incorporated at baseline, early during treatment, and at suspected progression to identify metabolic response or emerging resistant regions before structural changes become apparent. Clinical decision-making would require predefined thresholds demonstrating that hpMRI findings alter management beyond standard MRI, perfusion imaging, spectroscopy, or PET. Broader adoption will also depend on workflow feasibility, reimbursement, examination time, infrastructure costs, and formal cost-effectiveness analyses demonstrating that improved diagnostic confidence or earlier treatment adaptation offsets the expense of specialized equipment and personnel. Future clinical implementation will depend not only on development of novel metabolic probes but also on standardized acquisition protocols, harmonized kinetic modeling approaches, and multicenter validation that account for substrate delivery, membrane transport, enzymatic exchange, and T1-related signal decay.

## 4. Conclusions

In summary, hpMRI is an emerging non-invasive, safe, and versatile technique that offers the potential to greatly enhance the reach and clinical applications of conventional MRI spectroscopy. Extensive pre-clinical research has prepared the field for further expansion towards new molecular pathways, and early clinical studies have demonstrated acceptable short-term tolerability of hyperpolarized [1-^13^C]pyruvate in limited patient cohorts. The significant costs, as well as standardization and comparison of hpMRI with current neuroradiology approaches, will require additional time and investment. Beyond diagnosis and treatment monitoring, longitudinal hpMRI may reveal evolving metabolic phenotypes associated with tumor aggressiveness, therapeutic resistance, and recurrence, thereby providing clinically relevant prognostic information before structural progression becomes apparent. Although hpMRI remains promising for metabolic phenotyping and treatment assessment, broader clinical adoption will require regulatory approval, standardized multicenter workflows, greater hardware availability, trained personnel, cost-effectiveness evidence, and demonstration that hpMRI meaningfully improves patient management beyond established imaging methods.

## Figures and Tables

**Table 1:** Major metabolic alterations in brain tumors and corresponding hyperpolarized magnetic resonance imaging probes.

hpMRI Probe	Pathway	Known Metabolic Shift	Clinical Relevance	Development Stage and Key Considerations	Advantage	Key Limitation
[1-^13^C]Pyruvate	Glycolysis/Warburg effect [1,2,3,11,14,24,25] Oxidative phosphorylation [12,21,24,25,26,27] Pentose phosphate pathway [6,28,29,30]	↑ Lactate production, ↑ LDH	Aggressive phenotype, recurrence	Clinically translated; strongest safety and feasibility evidence, but standardized diagnostic thresholds remain unavailable.	Clinically mature; assesses glycolytic and oxidative metabolism.	Delivery-dependent; no validated thresholds.
[1-^13^C]Pyruvate → bicarbonate		↓ PDH flux, ↓ bicarbonate	Tumor progression		Reflects PDH-mediated oxidation.	Weak signal; model- and delivery-sensitive.
δ-[1-^13^C]-gluconolactone		↑ NADPH production	Redox buffering, radioresistance	Preclinical; targets G6PD/PPP activity and TERT-associated metabolism; requires human safety and delivery validation.	Measures G6PD/PPP activity.	Human feasibility unproven.
Hyperpolarized glutamine	Glutamine metabolism [3,31,32] IDH1 metabolism [5,33]	Glutamine addiction	Survival, therapy resistance	Preclinical; may assess glutamine dependence, but transport, rapid metabolism, and spectral complexity are challenges.	Assesses glutamine dependence.	Rapid metabolism and spectral overlap.
α-ketoglutarate probes		α-KG → 2-HG	Molecular classification	Preclinical/early translational; potentially specific for mutant IDH metabolism, but human sensitivity and reproducibility remain unestablished.	Detects IDH-mutant metabolism.	Limited human validation.
Gluconolactone	TERT-related metabolism [6,29,30] Redox metabolism [34,35,36]	↑ G6PD/PPP	Tumor aggressiveness		Measures G6PD/PPP activity.	Human feasibility unproven.
HP-[1-^13^C]NAC		↑ GSH dependence	Ferroptosis resistance	Preclinical; enables dynamic redox assessment, but interpretation may be affected by delivery and competing thiol reactions.	Assesses redox and glutathione biology.	Preclinical; delivery-dependent.
Acetoacetate probes	Ketone body metabolism [37,38]	Altered acetoacetate use	Metabolic flexibility	Preclinical; may characterize ketone body utilization and metabolic flexibility; limited brain tumor validation.	Evaluates ketone metabolism.	Limited tumor validation.
Branched amino acid tracers		↑ BCAT1 activity	IDH-wt aggressiveness	Preclinical; may reflect BCAT1-associated aggressive phenotypes; sensitivity and molecular specificity require validation.	May detect BCAT1-associated phenotypes.	Specificity and feasibility unknown.
Hyperpolarized [1,4-^13^C_2_]fumarate	Cell death and membrane integrity [39]	Fumarate-to-malate conversion after membrane disruption	Early treatment-response and necrosis imaging	Preclinical/early translational; potentially specific for treatment-induced cell death, but brain delivery, signal sensitivity, and human validation remain limited.	Detects treatment-induced cell death.	Preclinical; limited brain delivery.
Hyperpolarized ^13^C-urea	Perfusion and vascular delivery [21,40,41,42]	Non-metabolic distribution through the vascular compartment	Distinguishing substrate delivery from intracellular metabolism	Early clinical translation outside neuro-oncology; useful as a perfusion reference but provides no direct metabolic information and requires co-polarization and sequence optimization.	Measures perfusion and delivery.	No direct metabolic information.

Abbreviations: hpMRI, hyperpolarized magnetic resonance imaging; LDH, lactate dehydrogenase; PDH, pyruvate dehydrogenase; NADPH, nicotinamide adenine dinucleotide phosphate; IDH1, isocitrate dehydrogenase 1; α-KG, alpha-ketoglutarate; 2-HG, 2-hydroxyglutarate; TERT, telomerase reverse transcriptase; G6PD, glucose-6-phosphate dehydrogenase; PPP, pentose phosphate pathway; GSH, glutathione; HP-[1-^13^C]NAC, hyperpolarized [1-^13^C]N-acetylcysteine; BCAT1, branched-chain amino acid transaminase 1; IDH-wt, isocitrate dehydrogenase wild-type. ↑ indicates increased production, activity, or metabolic flux; ↓ indicates decreased production, activity, or metabolic flux; → indicates metabolic conversion from one metabolite to another.

**Table 2:** Summary of original studies employing hyperpolarized ^13^C magnetic resonance imaging (hpMRI) in brain tumors. Rows are color-coded according to probe type and study focus: Dark grey: ^13^C pyruvate-based probes and related glycolytic or oxidative metabolism studies. Light grey: Alternative ^13^C-labeled substrates (e.g., α-ketoglutarate, glutamate, acetoacetate, gluconolactone) used to interrogate specific metabolic pathways or genetic signatures. Light blue: Engineering, acquisition, or computational advances—new coils, pulse sequences, denoising, and kinetic-modeling approaches.

Authors	Title	Journal	Evidence Category and Cohort	Methods	Findings
Grist et al. [27]	Quantifying normal human brain metabolism using hyperpolarized [1–^13^C] pyruvate and magnetic resonance imaging.	*NeuroImage. 2019*	Healthy-volunteer, first-in-human feasibility study	Intravenously injected hyperpolarized [1–^13^C]pyruvate in the brain of healthy human volunteers for the first time.	In vivo probing of LDH and PDH by measuring [1–^13^C]pyruvate, [1–^13^C]lactate and [^13^C]bicarbonate.
Park et al. [23]	Development of methods and feasibility of using hyperpolarized carbon-13 imaging data for evaluating brain metabolism in patient studies.	*Magnetic resonance in medicine. 2018*	Early technical and patient-feasibility study	^13^C radiofrequency coils and pulse sequences tested in a phantom, dynamic sequences used in human patients.	Safety and feasibility of using hyperpolarized [1-^13^C]pyruvate to evaluate in vivo brain metabolism.
Miloushev et al. [9]	Metabolic imaging of the human brain with hyperpolarized ^13^C pyruvate demonstrates ^13^C lactate production in brain tumor patients.	*Cancer research. 2018*	Exploratory clinical study including untreated and recurrent brain tumors	First dynamically acquired human brain HP ^13^C metabolic spectra and spatial metabolite maps in cases of both untreated and recurrent tumors.	Production of HP lactate from HP pyruvate by tumors was indicative of altered cancer metabolism. Findings correlated with standard clinical brain MRI, MRI DCE perfusion, and FDG PET/CT.
Autry et al. [10]	Characterization of serial hyperpolarized ^13^C metabolic imaging in patients with glioma.	*NeuroImage: Clinical. 2020*	Longitudinal pilot study of three healthy volunteers and five patients with glioma	HP [1-^13^C]pyruvate MRI performed on 3 healthy volunteers and 5 patients s/p TMZ, XRT, anti-angiogenic/investigational agents.	*k*_PL_ and *k*_PB_ globally elevated following anti-angiogenic treatment, while disease progression showed elevated *k*_PL_ in Gd-enhancing and non-enhancing lesions.
Gordon et al. [45]	Translation of Carbon-13 EPI for hyperpolarized MR molecular imaging of prostate and brain cancer patients.	*Magnetic resonance in medicine. 2019*	Multi-organ technical translation study including patients with high-grade brain tumors	3T hpMRI in patients with prostate cancer and high-grade brain tumors, studied with hp [1-^13^C]pyruvate.	High pyruvate signal was seen throughout prostate and brain, as well as conversion to lactate. Bicarbonate production detected in the brain.
Mammoli et al. [46]	Kinetic modeling of hyperpolarized carbon-13 pyruvate metabolism in the human brain.	*IEEE Trans Med Imaging. 2020*	Technical kinetic-modeling study involving 10 brain tumor examinations	Hp [1-^13^C]pyruvate injected in 10 brain tumors to measure the conversion to lactate (k_PL_) and bicarbonate (k_PB_).	Input-less model had the best agreement with data. Post-fitting error criteria for voxel selection provided higher precision and spatial coverage.
Autry et al. [47]	Comparison between 8-and 32-channel phased-array receive coils for in vivo hyperpolarized ^13^C imaging of the human brain.	*Magnetic resonance in medicine. 2019*	Single-patient hardware comparison	Comparison between 8- and 32-channel receiver arrays in one patient with 1-^13^C]pyruvate hpMRI.	8-channel array has SNR benefits along lateral aspects; 32-channel array has greater coverage and uniform coil-combined profile.
Autry et al. [48]	Pilot Study of Hyperpolarized ^13^C Metabolic Imaging in Pediatric Patients with Diffuse Intrinsic Pontine Glioma and Other CNS Cancers.	*American Journal of Neuroradiology. 2021*	Pediatric safety and feasibility pilot study involving six patients with brainstem or other CNS tumors	HpMRI with [1-^13^C]-labeled pyruvate in 6 pediatric patients harboring brainstem tumors. Two dose levels used.	[1-^13^C]-labeled pyruvate well tolerated, bicarbonate and lactate visualized in the patient’s brain.
Autry et al. [25]	Multi-parametric hyperpolarized ^13^C/1H imaging reveals Warburg-related metabolic dysfunction and associated regional heterogeneity in high-grade human gliomas	*NeuroImage: Clinical. 2023*	Exploratory multiparametric study of 15 patients with high-grade glioma	Multi-parametric ^1^H/HP-^13^C pyruvate MRI acquired in 15 patients with high-grade glioma. Metabolic data were used to analyze contrast- and non-contrast-enhancing regions, as well as normal white matter.	Changes in perfusion and H choline-to-N-acetylaspartate index between different regions and tumor histologies.
Zaccagna et al. [24]	Imaging Glioblastoma Metabolism by Using Hyperpolarized [1-^13^C]Pyruvate Demonstrates Heterogeneity in Lactate Labeling: A Proof of Principle Study	*Radiology Imaging Cancer. 2022*	Proof-of-principle study of healthy volunteers and treatment-naïve patients with glioblastoma	^13^C pyruvate administered to healthy volunteers and GBM patients.	A lower bicarbonate:pyruvate ratio was found in the tumor. Tumor lactate and bicarbonate were correlated with pyruvate signal.
Autry et al. [49]	Advanced hyperpolarized ^13^C metabolic imaging protocol for patients with gliomas.	*Cancers (Basel). 2024*	Clinical protocol and repeatability study involving 42 patients and 100 glioma imaging sessions	Developed and implemented a standardized HP [1-^13^C]pyruvate MRI protocol across 100 imaging sessions in 42 glioma patients.	Demonstrated reproducible, clinically feasible imaging and kinetic modeling (k_PL, k_PB) with high repeatability and patient safety.
Cappelletto et al. [50]	Hyperpolarized ^13^C lactate-to-bicarbonate signal ratio predicts brain metastases response to stereotactic radiosurgery.	*Neuro-Oncology Advances. 2025*	Single-center observational study of 18 patients with 44 brain metastases	HP [1-^13^C]pyruvate MRI performed pre-SRS in 18 patients (44 brain metastases); lactate-to-bicarbonate ratio used as metabolic biomarker.	Elevated L/B ratio predicted poor response and local recurrence after SRS, outperforming conventional MRI markers.
Blazey et al. [51]	Spatial distribution of hyperpolarized [1-^13^C]pyruvate MRI and metabolic PET in the human brain.	*Imaging Neuroscience (Cambridge). 2025*	Healthy-volunteer multimodal imaging study	HP [1-^13^C]pyruvate MRI combined with FDG-PET in healthy volunteers to define normative metabolic distribution.	Identified reproducible regional pyruvate uptake and metabolic conversion patterns, establishing baseline reference data for brain tumor imaging.
Molloy et al. [52]	MR-detectable metabolic biomarkers of response to mutant IDH inhibition in low-grade glioma.	*Theranostics. 2020*	Preclinical cell-based IDH-mutant glioma study	IDH1mut-expressing lines, NHAIDH1mut and U87IDH1mut assessed with 1H and ^13^C hpMRI after treatment with IDHmut inhibitors.	Hydroxyglutarate (2-HG) was found to be decreased after treatment. Further, glutamine flow appeared to be affected by AG-120 and AG-881 use.
Batsios et al. [53]	Imaging telomerase reverse transcriptase expression in oligodendrogliomas using hyperpolarized δ-[1-^13^C]-gluconolactone	*Neurooncol Advances. 2023*	Preclinical molecular-imaging study of TERT-associated metabolism in oligodendroglioma	TERT is associated with upregulation of glucose-6-phosphate dehydrogenase (G6PD) in oligodendroglioma. δ-[1-^13^C]-gluconolactone was used to assess TERT expression in oligodendroglioma.	TERT silencing decreased 6-PG production from hp δ-[1-^13^C]-gluconolactone in oligodendroglioma cells. TERT rescue restored G6PD activity. No expression of G6PD was noticed in normal brain.
Vaziri et al. [54]	Assessment of higher-order singular value decomposition denoising methods on dynamic hyperpolarized [1-^13^C]pyruvate MRI data from patients with glioma	*NeuroImage: Clinical. 2022*	Technical post-processing study using dynamic glioma hpMRI data	Dynamic hp [1-^13^C]pyruvate MRI for higher-order singular value decomposition (HOSVD) denoising to enhance signal in glioma patients. Two HOSVD denoising techniques were tested.	Both techniques improved metabolite SNR and regional signal. More voxels with minimum SNR and maximum kinetic modeling error were achieved with new post-processing techniques in tumor lesions.

Abbreviations: hpMRI, hyperpolarized magnetic resonance imaging; GBM, glioblastoma multiforme; k_PL, apparent rate constant for pyruvate-to-lactate conversion; k_PB, apparent rate constant for pyruvate-to-bicarbonate conversion; LDH, lactate dehydrogenase; PDH, pyruvate dehydrogenase; SRS, stereotactic radiosurgery; ^18^F-fluorodeoxyglucose positron emission tomography; EPI, echo-planar imaging; SNR, signal-to-noise ratio.

**Table 3:** Comparison of hpMRI with conventional and advanced imaging modalities used in brain tumors. Diagnostic-performance estimates are representative values from individual studies and may vary according to cohort, reference standard, acquisition protocol, and threshold.

Modality	Principal Biological or Imaging Readout	Temporal Characteristics	Representative Diagnostic Performance	Current Clinical Utility and Limitations
Conventional contrast-enhanced MRI and T2/FLAIR [63]	Anatomy, tumor volume, edema, and blood–brain barrier disruption	Static anatomical images acquired over minutes	No universally validated standalone sensitivity or specificity for distinguishing recurrence from treatment effect	Standard for diagnosis, operative planning, radiation planning, and RANO-based surveillance. Enhancement is nonspecific and may be altered by corticosteroids, anti-angiogenic therapy, inflammation, pseudoprogression, and radiation necrosis.
Perfusion MRI: DSC/DCE [64]	Tumor vascularity, cerebral blood volume, cerebral blood flow, and vascular permeability	Dynamic acquisition during contrast passage, followed by quantitative maps	In a 40-patient study, sensitivity and specificity were 81.0% and 76.9% for cerebral blood volume, 77.3% and 84.6% for cerebral blood flow, and 61.9% and 80.0% for Ktrans. Combined perfusion accuracy was 82.5%.	Useful for distinguishing recurrent tumor from treatment-related change and identifying highly vascular regions. Results depend on acquisition, contrast leakage correction, normalization, and threshold selection.
Diffusion-weighted MRI/ADC [64]	Water mobility as an indirect marker of cellularity and tissue integrity	Diffusion maps acquired over several minutes	An ADC-ratio threshold of 1.27 differentiated recurrence from treatment-induced necrosis with 65% sensitivity and 100% specificity in one comparative study.	Widely available and does not require contrast. Interpretation may be confounded by necrosis, edema, hemorrhage, treatment effects, and intratumoral heterogeneity.
Proton magnetic resonance spectroscopy [65]	Steady-state concentrations of choline, N-acetylaspartate, creatine, lactate, lipids, and selected molecular metabolites such as 2-hydroxyglutarate	Spectral acquisition generally requires several minutes and does not directly measure rapid metabolic flux	In one study, the choline-to-creatine ratio showed 70% sensitivity and 78.6% specificity for recurrent glioma. Combining MRS with perfusion increased diagnostic accuracy from 82.5% to 90.0%.	Provides biochemical characterization and may support assessment of recurrence or IDH status. Limited by voxel size, spectral overlap, field heterogeneity, partial-volume effects, and technical expertise.
FDG-PET and amino-acid PET [66]	Radiotracer uptake, transport, and retention	Uptake and imaging generally occur over tens of minutes	In a 32-patient FET PET/MRI study, FET tumor-to-background ratio had 94.1% accuracy; combined PET/MRI parameters achieved 96.9% accuracy, 100% sensitivity, and 85.7% specificity for recurrence versus radionecrosis.	Amino-acid PET can improve recurrence assessment and tumor delineation. FDG-PET is limited by high physiologic brain uptake. PET requires ionizing radiation, tracer availability, and dedicated infrastructure.
Hyperpolarized ^13^C MRI [24,42,45]	Real-time substrate delivery and enzyme-mediated metabolic conversion, including pyruvate-to-lactate and pyruvate-to-bicarbonate flux	Dynamic human brain acquisitions have achieved approximately 3–4.3 s temporal resolution, with metabolic information collected over a short post-injection window	Sensitivity, specificity, diagnostic accuracy, and clinically validated thresholds have not yet been established in adequately powered brain tumor cohorts	Provides non-radioactive, pathway-specific metabolic flux and may detect biological changes before anatomical progression. Current limitations include small cohorts, short-lived signal, lower spatial resolution, specialized infrastructure, and limited multicenter validation.

## Data Availability

No new data were created or analyzed in this study. Data sharing is not applicable to this article.

## Abbreviations

The following abbreviations are used in this manuscript:

^1^H-MRS	Proton magnetic resonance spectroscopy
^13^C	Carbon-13
2-HG	2-hydroxyglutarate
5-ALA	5-aminolevulinic acid
BBB	Blood–brain barrier
BCAT1	Branched-chain amino acid transaminase 1
DCE	Dynamic contrast-enhanced
DIPG	Diffuse intrinsic pontine glioma
DNP	Dynamic nuclear polarization
EGFR	Epidermal growth factor receptor
EPI	Echo-planar imaging
FDG-PET	Fluorodeoxyglucose positron emission tomography
FGS	Fluorescence-guided surgery
GBM	Glioblastoma
GSH	Glutathione
G6PD	Glucose-6-phosphate dehydrogenase
HGG	High-grade glioma
HP	Hyperpolarized
hpMRI	Hyperpolarized magnetic resonance imaging
HP-[1-^13^C]NAC	Hyperpolarized [1-^13^C]N-acetylcysteine
IDH1	Isocitrate dehydrogenase 1
kPB	Apparent rate constant for pyruvate-to-bicarbonate conversion
kPL	Apparent rate constant for pyruvate-to-lactate conversion
LDH	Lactate dehydrogenase
MCT	Monocarboxylate transporter
MRI	Magnetic resonance imaging
MRS	Magnetic resonance spectroscopy
MYC (c-MYC)	MYC proto-oncogene, bHLH transcription factor
NADPH	Nicotinamide adenine dinucleotide phosphate
PDH	Pyruvate dehydrogenase
PET	Positron emission tomography
PpIX	Protoporphyrin IX
PPP	Pentose phosphate pathway
SNR	Signal-to-noise ratio
SRS	Stereotactic radiosurgery
TERT	Telomerase reverse transcriptase
TMZ	Temozolomide
TRI	Tensor rank truncation-image enhancement
GL-HOSVD	Global–local higher-order singular value decomposition
HOSVD	Higher-order singular value decomposition
XRT	Radiation therapy
α-KG	Alpha-ketoglutarate
